# Public Policies and One Health in Brazil: The Challenge of the Disarticulation

**DOI:** 10.3389/fpubh.2021.644748

**Published:** 2021-06-04

**Authors:** Isis de Freitas Espeschit, Clara Marques Santana, Maria Aparecida Scatamburlo Moreira

**Affiliations:** ^1^Laboratory of Bacterial Diseases, Preventive Veterinary Medicine and Public Health Sector, Veterinary Department, Universidade Federal de Viçosa, Viçosa, Brazil; ^2^Medical School, Universidade Federal de Juiz de Fora, Valadares, Brazil

**Keywords:** Health Surveillance, agricultural defense, animal health, animal-human bond, zoonosis

## Abstract

Working the One health strategy in developing countries is a challenge, due to structural weaknesses or deprivation of financial, human, and material resources. Brazil has policies and programs that would allow continuous and systematic monitoring of human, animal, and environmental health, recommending strategies for control and prevention. For animals, there are components of the Epidemiological Surveillance of zoonosis and Animal Health Programs. To guarantee food safety, there are Health Surveillance services and support of the Agropecuary Defense in the inspection of these products, productive environments, and their inputs. Environmental Surveillance Services monitor water and air quality, which may influence health. For human health, these and other services related to Health Surveillance, such as Worker Health and Epidemiological Surveillance, which has a training program responsible for forming professionals groups to respond effectively to emergencies in public health are available. Therefore, Brazil has instruments that may allow integrated planning and intervention based on the One Health initiative. However, the consolidation of this faces several challenges, such as insufficient resources, professional alienation, and lack of the recognition of the importance of animal and environmental health for the maintenance of human and planetary well-being. This culminates in disarticulation, lack of communication, and integration between organizations. Thus, efforts to share attributions and responsibilities must be consolidated, overcoming the verticality of the actions, promoting efficiency and effectiveness. Finally, this perspective aims to describe the government instruments that constitute potential national efforts and the challenges for the consolidation of the One Health initiative in Brazil.

## Introduction

One Health is an integrative and cooperative health initiative, with a transdisciplinary approach to health promotion and surveillance at the local, regional, national or global level, aiming to achieve optimal health conditions, given the recognition of the interconnection and interdependence between human, animals, and the environment (1, 2).

Historically, the perspective that environmental health affects humans has existed since the classical era. Hippocrates stated that biological and environmental conditions affect human health (3). Afterward, Aristotle and Galen sought to elucidate similarities between human and animal vital systems, allowing the creation of Comparative Medicine (4, 5).

In the 19^th^ century, pathologist Robert Virchow coined the term “zoonosis,” to name diseases transmitted from animals to humans, based on his observations with helminths, believing that there is no separation between animal and human health (5, 6).

Despite the recognition of this relationship, it was only 1970s that the epidemiologist Calvin Schwabe introduced the “One Medicine” theory, to name, in a holistic way, the connection between animals and humans in nutrition, habitat, and health, stating that both human and veterinary medicine have the same scope in physiology, anatomy, and pathology (7, 8).

The United Nations (UN), World Health Organization (WHO), Food and Agriculture Organization of the United Nations (FAO), and the World Organization for Animal Health (OIE) have recently established a cooperation to promote the perspective of One Health, encouraging the implementation of government policies and programs guided by it (9–11).

To this end, the Global Health Security Agenda (GHSA) was signed in 2014 with more than 64 nations and international non-governmental organizations supporting the collaborative approach. This will accelerate the consolidation and compliance of the requirements from the OIE Terrestrial and Aquatic Animal Health Codes, the WHO International Health Regulations (IHR), and other global health regulations for planetary health (8, 12, 13).

Despite the increase in rhetoric and support for the One Health model, it is difficult to find authentic examples of multidisciplinary or multi-sectoral efforts that transcend traditional public, animal, and environmental health “silos” (13, 14).

The ability to face threats to public and animal health effectively and efficiently requires effort and proper planning. A prerequisite for this is the training and capacitation of the public and private health sectors. However, this, within the One Health strategy, remains a challenge, especially for developing countries. This stems from competing priorities, insufficient and fragmented funding, with a lack of integration and communication between sectors (14, 15).

Some positive international experiences in this path have been reported, such as the One Health Workforce (OHW) project by the United States Agency for International Development (USAID). This aims to build partnerships with institutions and leaders in Africa and Southeast Asia to assist in the identification of technical, collaborative, and organizational characteristics, important for the development of the workforce in alignment with the goals established in the GHSA and by the Joint External Evaluation– International Health Regulations (JEE – IHR) (16, 17).

In Latin America, the incorporation of the One Health strategy into government plans is timid and the integration of animal, environmental and human programs and sectors is insignificant (9–11).

In Brazil, the One Health concept itself requires recognition by health professionals and is not acknowledged by important entities such as the Federal Council of Medicine. As an effect, the power of interventions and control strategies over the environmental causes of diseases is limited, and these activities are often restricted to veterinary professionals. On the other hand, public policies for Health Surveillance (HS) have allowed the gradual insertion of One Health supportive efforts in health practices, however with little integration between their organizations (9–11, 17, 18).

An adequate surveillance system, with a strong laboratory network, is the key component of any disease prevention and control strategy. To develop an effective One Health implementation plan, it is necessary to reexamine how the existing systems are structured, resourced, and managed. These analyzes would contribute to the development and sustainability of the synergy between human, animal, and environmental health initiatives (12–14, 19).

Thus, this perspective article aims to present the potential, successes and challenges of government health strategies, programs and policies in Brazil, from One Health point of view. The present discussion is based on the analysis of the official documents, legislation and health informational systems.

## The Potential Government Instruments for the Consolidation of One Health in Brazil

One of the most important aspects of pathogen control at the human, animal, and environmental interface is the development of appropriate scientific-based Surveillance and Risk Management policies that respect transboundary regulations (12, 14, 19).

In Brazil, there is a complex system for monitoring human, animal, and environmental health conditions, based on the Health Surveillance System (HSS) and in the use of epidemiology as a planning tool. This system is supported by organizations such as the Ministério da Agricultura Pecuária e Abastecimento- MAPA (Ministry of Agriculture, Livestock and Supply) and the Sistema Único de Saúde- SUS (Unified Health System) and its respective policies, programs, regulations, and health information systems as summarized in Tables 1, 2 and described in detail in the following text (46–49).

**Table 1:** Organizational components of Health Surveillance System (HSS) programs and policies regarding the human, animal, and environmental health.

**Acronym**	**Program**	**Regulations**
**HUMAN AND ANIMAL HEALTH SURVEILLANCE**		
**ESS**	Epidemiological Surveillance System Zoonosis Epidemiological Surveillance System	Ordinance No. 2,254, OF AUGUST 5, 2010 Institutes Epidemiological Surveillance in Hospital Settings defines the competencies for the Union, the States, the Federal District, the Municipalities, the criteria for the qualification of the national reference hospital units and defines the scope of the activities to be developed by the Hospital Centers of Epidemiology (20). LAW No. 6,259, OF OCTOBER 30, 1975. Provides for the organization of Epidemiological Surveillance actions, the National Immunization Program establishes rules regarding the compulsory notification of diseases and provides other measures (21). NATIONAL COUNCIL OF HEALTH SECRETARIES-TECHNICAL NOTIFICATION, 02 / 2014- 2 definition of health actions and services aimed at surveillance, prevention, and control of zoonosis and accidents caused by venomous and poisonous animals, of relevance to public health (22).
**EpiSUS**	Training program in Applied Epidemiology to the Services of the Unified Health System	MINISTRY OF HEALTH - Ordinance No. 1,430, OF JUNE 11, 2018- Amends Consolidation Ordinance No. 5, of September 28, 2017, to institute the Training Program in Epidemiology Applied to the Services of the Unified Health System - EpiSUS Program (23).
**OHS**	Occupational Health Surveillance	MINISTRY OF HEALTH - Ordinance No. 1,823, OF AUGUST 23, 2012-Institutes the National Policy for Workers' Health (24). MINISTRY OF HEALTH. Ordinance No. 1339. 1999 November 18. To institute the List of Work-related Diseases, to be adopted as a reference for injuries originated in the work process in the Unified Health System, for clinical and epidemiological use, contained in Annex I of this Ordinance (25).
**ENVIRONMENTAL HEALTH SURVEILLANCE**		
**VIGIÁGUA**	Water Quality Monitoring Program	Ordinance No. 2,914 of December 12, 2011, provides for the control and surveillance procedures of the quality of water for human consumption and its drinking water standard.
**VIGIPEQ**	Health Surveillance of Populations Exposed to Chemical Contaminants	Ministry of Health of Brazil. National health surveillance program for populations exposed to contaminated soil. 2007. Brasília (26).
**VIGISOLO**	Health Surveillance of Populations Exposed to Contaminated Soil	Normative Instruction MS No. I of March 7, 2005 - Regulates Ordinance No. 1,172 / 2004 / GM, concerning the competencies of the Union, states, municipalities, and the Federal District in the area of environmental health surveillance (27).
**VIGIDESASTRES**	Environmental Health Surveillance related to Natural Disasters	NATIONAL HEALTH FOUNDATION. Guide to environmental health surveillance / National Health Foundation. 2002. Brasília (28).
**VIGIAR**	Air Quality Monitoring Program	

*These components are part and subject to the national Unified Health System (SUS- Sistema Único de Saúde)*.

**Table 2:** Organizational components of Health Surveillance System (HSS) programs and policies regarding the human, animal, and environmental health in Brazil.

**Acronym**	**Program**	**Regulations**
**MAPA- MINISTÉRIO DA AGRICULTURA, PECUÁRIA E ABASTECIMENTO (MINISTRY OF AGRICULTURE, LIVESTOCK AND SUPPLY)**		
**VIGIAGRO**	Agropecuary Surveillance/defense	Law 8,171, of January 17, 1991 - Provides for agricultural policy (29). Decree n° 30.691, of March 29, 1952 -Institutes the Regulation of Industrial and Sanitary Inspection of Products of Animal Origin and obliges MAPA to inspect animals and derived products in the sea and river ports and border posts. Institutes the Inspection Regulation Industrial and Sanitary Products of Animal Origin and obliges MAPA to inspect animals and derived products in sea and river ports and border posts (30). Law No. 6,198, of December 26, 1974 - Forces MAPA to inspect products for animal feed in ports and border posts. Forces MAPA to inspect products for animal feed in ports and border posts (31). Law No. 7,802, of July 11, 1989 - Forces the Federal Government to inspect the import of pesticides (32). Law No. 7,678, of November 8, 1988 - Provides for the production, circulation, and commercialization of wine and grape and wine derivatives, and makes other provisions (33). Law No. 8,918, of July 14, 1994 - Provides for the standardization, classification, registration, inspection, production, and inspection of beverages, authorizes the creation of the Intersectorial Beverage Commission, and makes other provisions (34). Decree No. 24,114 of April 12, 1934. - Organizes the Unified System of Attention to Agricultural Health and makes other provisions (35). Normative Instruction No. 51, of November 7, 2011 (36). Normative Instruction No. 91, of September 18, 2020 - Provides for the Use of the LPCO Module in Import Operations for Products of Agricultural Interest (37). Annex IN 51/2011 - NCM list of products that will meet the regulatory criteria and procedures for inspection, inspection, quality control, and risk analysis systems, established by the Ministry of Agriculture, Livestock and Supply (MAPA) (36).
**AHP**	Animal Health Programs, namely: National Equine Health Program National Poultry Health Program	MAPA - Normative Instruction No. 6, of January 16, 2018, institutes the National Equine Health Program (38). MINISTERIAL ORDINANCE No. 193, SEPTEMBER 19, 1994- Establishes the National Poultry Health Program within the scope of the DSA and creates the Consultative Committee of the Poultry Health Program (39).
	National Program for the Control and Eradication of Brucellosis and Tuberculosis	MAPA - Normative Instruction 02/2001 Normative Instruction SDA n° 10/2017, to reduce the negative impacts of Brucellosis and Tuberculosis on human and animal health (40).
	National Goat and Sheep Health Program	MAPA - Normative Instruction No. 87, of December 10, 2004. Institutes the National Program for the Health of Goats and Sheep (41).
	National Herbivore Rabies Control Program	MAPA - Normative Instruction No. 5 01/03/200, Approves the Technical Norms for the control of rabies of domestic herbivores (42).
	National Apiculture Health Program	MAPA - Normative Instruction No. 16, of May 8, 2008, institutes the National Program for Beekeeping Health (PNSAp) (43).
	National Program for the Eradication and Prevention of Foot-and-Mouth Disease,	MAPA - Normative Instruction No. 44 of 10/02/2007 Approves the general guidelines for the Eradication and Prevention of Foot-and-Mouth Disease, to be observed throughout the National Territory, with a view to the implementation of the National Program for the Eradication and Prevention of Foot-and-Mouth Disease (PNEFA), like the one established by the Unified Agricultural Health Care System (44).
**ANVISA- AGÊNCIA NACIONAL DE VIGILÂNCIA SANITÁRIA-(NATIONAL AGENCY OF SANITARY SURVEILLANCE)**		
	Sanitary Surveillance System	Law No. 9,782, of January 26, 1999, institutes the National Health Surveillance Agency, which is responsible for ensuring the safety and quality of goods, products, environments, inputs, and services that can convey risks to individual or collective health (45).

Brazil's health system emerged due to an organizational restructuring and popular social movements. With the implementation of this universal and integral system, normative actions created for stimulation of the HSS, brought with it the redefinition of health practices, allowing their greater integration with epidemiology. This surveillance system is based on four main pillars, focused mainly on conditions that affect human health, namely: Epidemiological, Sanitary, Environmental and Occupational Health Surveillance Systems (12–15, 50).

The National Environmental Health Surveillance System performs continuous and systematic monitoring of environmental conditions that may interfere with human and animal health such as water, soil, air, biological factors and vectors, environmental disasters, and accidents with contaminants. The operational instruments to make this viable are VIGIÁGUA (evaluates the quality of water for human consumption), VIGIPEQ (acts on environmental contaminating chemicals), VIGISOLO (acts on the quality of soil and cultivation activities), VIGIAR (evaluates air quality), and VIGIDESASTRES (surveillance and management of environmental disasters) (26–28, 46–48).

The Epidemiological (ES) component of the Surveillance System is responsible for systematic monitoring of adverse health events, and control measures proposition, consolidated by the National ES System and its sub-areas, allowing significant advances in the capacity to respond to health problems. ES monitors communicable and non-communicable diseases and other health problems, such as accidents, violence, and a strategic area, that performs Zoonosis Surveillance, recognizing the importance of the animal-human bond. The latter is responsible for monitoring the progress of animal diseases and accidents with venomous animals. The recognition of the importance of zoonotic diseases by the health system is extremely relevant; both for management and for the health care itself, since, on average 60–80% of human diseases are of animal origin (20–25, 49, 51–53).

In the context of ES, Brazil also has a Training program in Applied Epidemiology to the Services of the Unified Health System- EpiSUS, part of the Department of Surveillance of Communicable Diseases of the Health Surveillance System. This program counts with multidisciplinary teams, aiming to enhance the ability to respond to public health emergencies and encourage the exchange of professionals and experiences at national and international levels, to improve the national technical capacity in field epidemiology, becoming an international reference in this field (23, 51, 53–57).

The Occupational Health Surveillance, part of HSS, monitors occupational risk factors and determinants of human health conditions that are related to the production and labor processes, as well as the ones related to the work environment (23, 54–57).

The Sanitary Surveillance System, coordinated by ANVISA, the national agency for Sanitary Surveillance, works in the regulation, control, and inspection of goods, products, and environments that are directly or indirectly related to health conditions, from production to consumption. It is recognized as a set of integrated institutional, administrative, programmatic, and social strategies, guided by public policies that aim the elimination, reduction, or prevention of individual and collective health risks, based on comprehensive services and actions that are essential to the health defense and promotion. This breadth of activities justifies the fact that these actions are developed as a democratic and participatory exercise and in an articulated way, to guarantee the quality of products, services, and environments, fundamental aspects for global health. Therefore, the Sanitary Surveillance is not restricted to purely technical actions, but its driving axes are actions aimed at the strengthening of the society and citizenship to promote health and preventing risks, damages or injuries (15, 45, 49, 54, 55, 58).

Also responsible for One Health, regarding animal health, food safety, trade and transit of products of animal and vegetable origin are the programs subjected to MAPA, the institution responsible for the National Agropecuary Defense (18, 57, 58).

The Agropecuary Defense relates to all stages of agricultural and livestock production, since the registration and inspection of agricultural inputs; production, industrialization, inspection and trade of products and by-products of animal and vegetable origin; besides acting in the import and export of inputs and products, as well as in the transit of these products and animals. In this way, it would not only promote and protect animal, human and environmental health but also help to increase economic incentives by promoting security and adding value to agricultural products (12, 29–36, 59–63).

Also subject to the National Agricultural Defense services, are the Animal Health Programs, namely: National Equine Health Program, National Poultry Health Program, National Program for the Control and Eradication of Brucellosis and Tuberculosis, National Goat and Sheep Health Program, National Herbivore Rabies Control Program, National Apiculture Health Program, National Program for the Eradication and Prevention of Foot-and-Mouth Disease, coordinated by MAPA (38–44, 61–64).

These programs aim to prevent, control, or eradicate diseases of public health importance or that may threaten international trade, ensuring animal health through health education activities; epidemiological studies; the inspection of breeding establishments, fiscalization of agricultural events and animal transit, notifying the occurrence of these diseases, in addition to guaranteeing the competitive value of the products in the international market (38–44, 61–64).

Furthermore, an operational instrument for Agropecuary Defense is VIGIAGRO, MAPA's instance that regulates the flow of animals, products, and agricultural inputs in frontiers, to ensure their safety and quality, preventing or minimizing potential risks to the One health (14, 18, 64–66).

In this construct, the programs and services presented have the potential for the insertion of the multidisciplinary and integrative approach of One Health, with intra and intersectoral collaboration Tis approach is appropriate to the current context of growing concerns about the consequences of the interactions between humans, animals, and the environment, in a productive framework based on capitalist ideals (58, 59, 67–69).

## Discussion

The importance of the policies and operationalization referred in this perspective article is acknowledged, but several challenges are found for the consolidation of its potential, the main ones being the disarticulation, the verticality of the actions, and the overlapping of attributions (1–5).

The central goals of the One Health model include altering the organization's work from a vertical and hierarchical approach to a horizontal and integrated one, in addition to moving from individual “silos” to a transdisciplinary functioning (1, 2, 14).

The difficulties in achieving these goals and in the integration between the spheres that work animal, human, and environmental health start in the professional training of those who work in these fields. Few individuals enter the workforce with crosscutting skills to thrive in transdisciplinary or multi-sectoral teams (14, 15).

The increase in the number and distribution of qualified personnel has proved to be one of the main limitations in most developing regions. This limitation, added to the financial, structural, and bureaucratic constraint faced by this sector, prevents it from satisfactorily exercising its potential, putting global health at risk (15).

The need and benefits of an integrated surveillance system to better understand the emergence and epidemiology of diseases have been demonstrated. This should count with a health care notification system and a comprehensive national database including environmental monitoring, human and animal health diagnostic systems, essential components of an integrated surveillance system, and for the implementation of One Health (12–15, 61).

Brazil has all these components, but they do not articulate or communicate. Even more, they sometimes overlap their attributions and compete for authority and hierarchy. The information produced by different organizations, rarely crosses that 'silo' that produced them, even though it could support health actions as a whole, producing and using information and communication in a shared way, to better instrumentalize the intervention (15).

For example, information on the incidence and prevalence of work-related illnesses could be used to modify production processes, minimizing the risks and potential damage. The occurrence of water and foodborne diseases could contribute to the improvement of the performance of Agropecuary Defense, among others. Just as, information about environmental health can be useful to guide all production processes, to help understanding the epidemiology and natural history of diseases, allowing the intervention in their origins. This would promote cheaper, more assertive, and definitive solutions to health problems and demands, making individuals, animals, and the environment healthier, more productive, and sustainable (12–15).

While some progress has been done, there is a need for continued investment and political commitment to address the persistent challenges in public health. As it can be seen in the Tables 1, 2, the programs are not recent efforts and some of them are more than 20 years old. This is also a challenge since they are sealed in their 'silos', without undergoing major changes over the years and without communicating with other programs and institutions. Still in this sense, it is clear that changes in global initiatives and situations such as the pandemic due to the coronavirus have little or no influence on the construction of regulatory frameworks.

The One Health initiatives are of complex consolidation from both a political, technical, and sanitary perspective (12, 14, 15). The implementation of the strategies presented here, associated with the complex movement of political and administrative restructuring in Brazil represented progress in the direction of producing more effective interventions, however, it further aggravated the fragmentation and sectorization of health actions. This resulted in a surveillance based primarily on a routine centered on the notification and investigation of cases and the transmission of data to other levels for the consolidation of bases and information systems, frequently not reaching the final goal of triggering an intervention (15).

The lack of articulation and use of health information for planning interventions can be evidenced with a contemporary example. The COVID-19 pandemic and the recent worsening of it at the national level in early 2021 are the results of a lack of planning and intervention by governmental health spheres that have proved inefficient and non-resolute. Instead of applying the information, fees, numbers to equip their infrastructure and instruct their professionals, the highest governmental spheres have chosen to hide and fragment the health information consolidated by the Epidemiological Surveillance Systems and not apply it for planning and structuring measures to mitigate the health crisis (70–72).

The integration and articulation between health actions, organizations, and policies would promote greater effectiveness and efficiency of health interventions, promoting global health and expanding the response capacity. However, there are still restrict limits to the autonomy of federal entities in Brazil, perpetuating an excess of verticality in programs and decisions, which makes it difficult to move toward the integration of surveillance systems in the perspective of democratizing the health practices (15, 59, 73).

The action of these entities and services should be in the sense of sharing attributions and responsibilities, without leaving behind the technical specification of each of the areas. This would imply new roles, as well as new dynamics, relationships, and innovative practices at all levels, which denotes the complexity and challenge of its implementation (59, 73, 74).

Thus, it is proposed to work, from the One Health approach, in an articulated and integrated set of actions, which assume specific configurations according to the environmental, animal, and human health situation of each territory, transcending the institutionalized spaces of the health services system, the verticality, and hierarchy of agencies and programs. Thus, seeking a dialogue between “cause control,” “risk control” and “damage control” through the redefinition of the object, the means of work, activities, and technical and social relations (12, 13, 15).

## Data Availability Statement

The original contributions presented in the study are included in the article/supplementary material, further inquiries can be directed to the corresponding author/s.

## Author Contributions

The authors contributed equally to all phases of the manuscript construction and revision.

## Conflict of Interest

The authors declare that the research was conducted in the absence of any commercial or financial relationships that could be construed as a potential conflict of interest.
